# Making adjustments to event annotations for improved biological event extraction

**DOI:** 10.1186/s13326-016-0094-9

**Published:** 2016-09-16

**Authors:** Seung-Cheol Baek, Jong C. Park

**Affiliations:** 1 Department of Computer Science, KAIST, 291 Daehak-ro, Daejeon, Republic of Korea; 2 Agency for Defense Development, Daejeon, Republic of Korea

## Abstract

**Background:**

Current state-of-the-art approaches to biological event extraction train statistical models in a supervised manner on corpora annotated with event triggers and event-argument relations. Inspecting such corpora, we observe that there is ambiguity in the span of event triggers (e.g., “transcriptional activity” vs. ‘transcriptional’), leading to inconsistencies across event trigger annotations. Such inconsistencies make it quite likely that similar phrases are annotated with different spans of event triggers, suggesting the possibility that a statistical learning algorithm misses an opportunity for generalizing from such event triggers.

**Methods:**

We anticipate that adjustments to the span of event triggers to reduce these inconsistencies would meaningfully improve the present performance of event extraction systems. In this study, we look into this possibility with the corpora provided by the 2009 BioNLP shared task as a proof of concept. We propose an Informed Expectation-Maximization (EM) algorithm, which trains models using the EM algorithm with a posterior regularization technique, which consults the gold-standard event trigger annotations in a form of constraints. We further propose four constraints on the possible event trigger annotations to be explored by the EM algorithm.

**Results:**

The algorithm is shown to outperform the state-of-the-art algorithm on the development corpus in a statistically significant manner and on the test corpus by a narrow margin.

**Conclusions:**

The analysis of the annotations generated by the algorithm shows that there are various types of ambiguity in event annotations, even though they could be small in number.

## Background

Current state-of-the-art approaches to biological event extraction train statistical models in a supervised learning manner on annotated corpora, where *event triggers*, or the expressions indicative of events, and *event-argument relations*, or relations between events and their participant, are annotated (e.g., [1, 2]). The readers are referred to [3] if the tasks are not familiar. Inspecting such corpora, we observed some cases where there is residual ambiguity in the span of event triggers (e.g., “transcriptional activity” vs. ‘transcriptional’). Because of the ambiguity, these gold-standard corpora would manifest inconsistencies across the span of event triggers. That is, there would be similar phrases where the span of their counterparts of event triggers is differently annotated, and as a result, such event triggers are syntactically characterized in a different way, suggesting a possibility that a statistical learning algorithm is hard to generalize from such event triggers that are similar, but differently annotated in a training corpus. We anticipate that adjustments to event annotations to reduce such inconsistencies would lead to a meaningfully improved performance of even the state-of-the-art event extraction systems. In this study, we look into this possibility with the corpora provided by the 2009 BioNLP shared task [3]. We note that this paper reports an extension of our previous work [4] with detailed discussions and more experimental results.

For example, consider sentence (1) from the training corpora, where the annotated event triggers are set in bold-face.

1. ... **express** either **decreased** or **increased numbers** of VDR. (pmid:9783909)

**Fig. 1:**
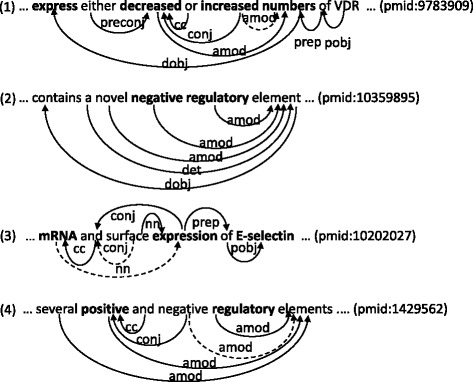
Dependency Graphs of Example Sentences. The graphs are basic Stanford dependency analyses by the Charniak-Johnson parser with a self-trained biomedical parsing model. In (*1*) and (*4*), dashed arrows indicate inferred dependency relations based on conjunctions. In (*3*), dashed arrows indicate that corresponding dependency relations are naturally expected dependency relations, but are missed in the analysis generated by the parser

The phrases ‘decreased’ and ‘increased numbers’ are annotated as event triggers of Negative and Positive Regulation events, respectively, that take a Gene Expression event with the event trigger ‘express’. These annotations are justifiable with respect to the meaning of these phrases, but there are alternatives, including one where the phrase ‘increased’ becomes the trigger of the Positive Regulation event. Despite the semantic similarity between these two events, their event-argument relations to the Gene Expression event are syntactically different (Fig. 1), in that the event trigger ‘decreased’ is the adjectival modifier (AMOD) of the direct object (DOBJ) of the phrase ‘express’, while the event trigger ‘increased numbers’ is the direct object (DOBJ) of the phrase ‘express’. However, if these event triggers are slightly adjusted, for example by dropping the word ‘numbers’ from the event trigger ‘increased numbers’, these event triggers and event-argument relations will come to have similar to share the similarity also in syntactic characteristics with respect to phrasal categories and shortest dependency paths. The inconsistencies would provide a valuable opportunity for improving the performance of event extraction, but the current state-of-the-art approaches have not seriously addressed them yet.

Note that one may still find that sentence (1) indicates a Regulation event, not these Positive and Negative Regulation events, but we can leave the identification of the Regulation event to an inference engine that would be deployed after event extraction systems, since the exact nature of an event can be inferred from the disjunction of these Positive and Negative Regulation events indicated by the syntactic construction “either A or B”.

The only reported effort would be to normalize multi-word event triggers into single-word event triggers with the help of the *Head-Word* rule, or a rule of taking the syntactic head word of an event trigger (e.g., [2, 5]), even though the rule often makes an apparently bad choice, as in the example above, where it picks out the constituent word “numbers” from the event trigger “increased numbers”, which does not have any meaning relevant to Positive Regulation events, and furthermore, is inconsistent with the event trigger “decreased”.

In this paper, as a proof-of-concept study, we examine the benefits of reducing inconsistencies across event annotations as follows. First, we use the Expectation-Maximization (EM) algorithm with Viterbi approximation, where latent variables encode events. Our experimental results show that the unmodified EM algorithm is defeated by our baseline algorithm, which is a learning algorithm that successfully trained state-of-the-art event extraction systems [2], in part because the EM algorithm adjusts the models to extract similar but unintended events. To overcome this problem, we use a posterior regulation technique of consulting the gold-standard annotations in the form of constraints. We come up with four constraints on the possible event annotations to be explored by the EM algorithm. The resulting algorithm, to be called the *informed EM* algorithm, turns out to outperform our baseline algorithm on the development corpus in a statistically significant manner (*p* −*value*=9.59 E-12) and on the test corpus by a narrow margin (51.6 % vs. 51.3 %). Thus, we found it beneficial to make proper adjustments to event trigger annotations. An analysis of the annotations generated by the algorithm shows that there are various types of ambiguity in event annotations including ambiguity in the span of event triggers, even though the algorithm finds only a small number of such cases.

To the best of our knowledge, this would be the first study where adjustments to the gold-standard annotations are made, even though there are NLP studies on a similar use of posterior regularization techniques including the one by Okita and colleagues [6], where partial annotations of alignment links are incorporated as prior knowledge into the word alignment process.

The rest of this paper is organized as follows. This Background section ends with the following short subsection “Biological Event Extraction Task”, which defines the task of biological event extraction. The Methods section develops our statistical models and learning algorithms. The Results section presents and analyzes experimental results. The Conclusion section presents possible future research directions and concludes this paper.

### Biological event extraction task

As a case study, we addressed the event extraction task as defined in the 2009 BioNLP shared task 1 [3], which was later renamed as GENIA Event Task 1 and extended to cover full papers in the 2011 BioNLP shared task [7], where biological events are used to refer to the changes of a state of one or more biological macromolecules. The task is to extract structured information on events from sentences in the biological literature, which consists of their event type and participants encoded with a controlled vocabulary that consists of nine event type terms (e.g., Gene Expression) and two role type terms (i.e., THEME and CAUSE).

The nine event types are divided into three groups according to their participants. The first group is *plain protein-taking events* that must take a single protein as THEME (e.g., Gene Expression). The second one is *multiple protein-taking events*, or events that take one or more proteins as THEME (e.g., Binding events). The third one is *event-taking events* that must take a single protein and event as THEME and may take a single protein and event as CAUSE (e.g., Positive Regulation and Negative Regulation). The events of the first group may be viewed as binary relations between event triggers and protein mentions, but those of the last two groups are different from binary relations, in that multiple protein-taking events take more than one argument and event-taking events allow nested event structures. Thus, the extraction of events poses challenges other than those of the extraction of binary relations, which have been extensively studied in the biomedical information extraction community.

## Methods

Following Björne and colleagues [5], we viewed the event extraction task as constructing directed graphs, where event triggers and event-argument relations are encoded with labeled nodes and edges, respectively. We constructed these directed graphs with the help of various resources including syntactic analyses. In this section, we first describe these resources used in our event extraction system and then develop graph representations, statistical models and learning algorithms, in this order.

### Resources

We used lexical and syntactic analyses to encode tokens and the relation between tokens into statistical models. As for lexical analyses, we used the baseforms and part-of-speech (POS) tags of the tokens included in the analyses by the Enju parser, which are available in the official website of BioNLP shared tasks (http://weaver.nlplab.org/~bionlp-st/BioNLP-ST/downloads/support-downloads.html). As for syntactic analyses, we use basic Stanford dependency analyses by the Enju parser with the GENIA model [8] together with those by the Charniak-Johnson parser [9] with a self-trained biomedical parsing model [10], since the Enju parser fails to generate analyses for a few sentences. These syntactic analyses are also available in the official website of BioNLP shared tasks.

As for protein mentions, we used their gold-standard annotations available on the official website of BioNLP shared tasks, which were given to the participants in the BioNLP shared tasks. The annotations contain multi-word protein mentions. Since most of them correspond to syntactic units (i.e., single words and phrases), we can easily combine tokens in multi-word protein mentions into single tokens and redirect their dependency relations.

Following Miwa and colleagues [1] and Kilicoglu and Bergler [11], we developed an *event trigger lexicon* for each event type for the purpose of identifying apparently incorrect candidates for event triggers as follows. Constituent words within annotated event triggers in the training corpus are scanned one by one. Each scanned constituent word is put into the lexicon that corresponds to the type of events anchored at the event trigger. When the constituent word contains hyphens, it is split by hyphens and the resulting components of the word are put into the lexicon together with the original constituent word. In a similar manner, we also constructed the stemmed version of each event trigger lexicon using Porter Stemmer.

The automatically constructed lexicons would contain a number of entries not helpful for checking if a token is part of an event trigger. To identify and remove such entries, we computed the *reliability scoreR*_*w*,*e*_ of each entry *w* in the lexicon for each event type *e*, as defined by Kilicoglu and Berger [11]:

1 $$ R_{w,e}={C_{w,e} \over C_{w}}  $$

where *C*_*w*,*e*_ is the number of times the entry *w* appears within the event trigger of events of type *e* and *C*_*w*_ is the number of times the entry *w* appears. Finally, we removed entries with reliability scores below 1 %.

After this removal, these lexicons still have either a part or the whole of 98 % of the annotated occurrences of event triggers in the training corpus, and can be used to identify candidate pairs of words *w* and event types *e* indicating that *w* might be part of an event trigger for event type *e*, where around 11 % of them are actually part of annotated event triggers.

### Graph representations

Let us consider how to encode multi-word event triggers. We came up with the following four possible forms of multi-word event triggers and manually searched the training corpus for cases corresponding to each possibility with the help of syntactic analyses by the Charniak-Johnson parser [9] with a self-trained biomedical parsing model [10], as shown in Fig. 1.

The first is that some event triggers are inherently multi-word expressions, as exemplified in (2), where words within the bold-faced event trigger “negative regulatory” of a Negative Regulation Event fully describe the nature of the event only together each other:

(2) ... contains a novel **negative regulatory** element... (pmid:10359895)

Second, some words in multi-word event triggers are adjacent to one another, but have no dependency relations among them, suggesting that at least the first and last words of each event trigger should be marked. Returning to sentence (2), the two words ‘negative’ and ‘regulatory’ are adjacent to each other and have no dependency relations between them in the generated dependency graph.

The third is that some words within multi-word event triggers are not consecutive to one another but have dependency relations among them, suggesting that dependency relations combining words within event triggers should be encoded. As an example, consider sentence (3), where the bold-faced word ‘expression’ is annotated as the trigger of Transcription and Gene Expression events, which produce the mRNAs and proteins of the gene E-selectin, respectively.

(3) ... **mRNA** and surface **expression** of E-selectin.... (pmid:10202027)

Our intuition is that the word ‘expression’ in combination with the word ‘mRNA’ describes the nature of Transcription events more fully than the word ‘expression’ alone, but only that the words ‘and’ and ‘surface’ appear in-between. That is, the words ‘mRNA’ and ‘expression’ in sentence (3) are not consecutive, but have a dependency relation NN between them.

Fourth, some words in multi-word event triggers might not be consecutive to one another and might not have dependency relations among them either. The effort to manually find such a case was not successful but we found a similar case. In sentence (4), the words ‘positive’ and ‘regulatory’ indicate together the presence of Positive Regulation events (not annotated on the training corpus), but these two words are not consecutive to each other and are not linked to each other through dependency relations in the generated dependency graph where these two words have the dependency relation AMOD to ‘elements’.

(4) ... several **positive** and negative **regulatory** elements.... (pmid:1429562)

Since these four different types of multi-word event triggers would make it complicated to represent the span of event triggers in the graphs, and since our focus here is not on exactly identifying the span of event triggers, we mark only single words within event triggers and encode the context of these marked words into statistical models to exploit other words within the span of event triggers in sensing the presence of the event triggers including them. For example, we may mark the word ‘regulatory’ as the anchor word of the event trigger “negative regulatory” in sentence (2) and encode its contextual features including the fact that the word ‘regulatory’ is adjacent to the word ‘negative’.

One natural candidate for words to be marked is the constituent words of an event trigger that we can use to encode syntactic relations between the event trigger and other words since we need them anyway, but this decision did not help to uniquely determine which word should be marked. Another conceivable decision, to be pursued in this article, is that a marked word can be used in describing as many syntactic relations between event triggers and participants as possible so that it is possible to easily find regularities in these syntactic relations only from a small number of instances. Henceforth, we will call such words meeting these decisions and being marked as **anchor words**. Of course, the choice of anchor words would be dependent on the way for describing syntactic relations between words and the training corpus, but there are predictable characteristics of anchor words.

First, when an event trigger corresponds to a phrase (e.g., the first and third observations above), the natural choice for the anchor word of the event trigger would be the head word of the phrase, since the dependency paths between the head word and words outside the phrase do not have other constituent words in the event trigger so that the located dependency paths can be used for different event triggers with the same head words. As a result, in sentence (3), ‘expression’ is preferable to ‘mRNA’.

Second, when an event trigger does not correspond to a phrase (e.g., the second and fourth observations above), the natural choice for the anchor word of the event trigger would be the word frequently occurring in various event triggers for the same event type. Since seven Positive Regulation event triggers contain ‘positive’ in the training corpus but only one Positive Regulation event trigger (‘positive regulatory role’) contains ‘regulatory’, ‘positive’ is preferable to ‘regulatory’ in sentence (4).

Now let us define the desirable output labels of tokens in the training corpus for trigger identification. All the words except for anchor words will be given the label ‘negative’. Anchor words will be labeled with more than one event type, since some event triggers indicating two different types of events share an anchor word as shown in sentence (3), where the word ‘express’ would be preferable anchor words for Transcription and Gene Expression events.

When turning to the label of edges, a question arises whether edges can be labeled with more than one role type, that is, whether an event takes a protein or another event both as THEME and CAUSE. To answer this question, we constructed graphs for sentences in the training corpus of 800 annotated abstracts with the Head-Word rule. There are only six edges labeled with more than one role (of around 8,200 edges labeled with one or more roles), suggesting that they are likely to be annotation noise. As a result, we allow edges to be labeled with at most one role type.

**Fig. 2:**
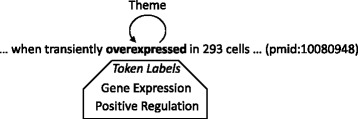
Event Graph with a Loop

The issues we have discussed so far are also relevant to relations, but there are issues specific to events, including the one that graphs with cycles and loops may lead to an infinite number of event-taking events with distinct event participants. As an example, consider Fig. 2, where the word in bold-face is the annotated event trigger of a Gene Expression event and a Positive Regulation event that takes the Gene Expression event as THEME. It would be straightforward to derive these Gene Expression and Positive Regulation events from the graph. The problem is that there is no principled way to rule out another Positive Regulation event with the derived Positive Regulation event as THEME.

One way is to disallow graphs with cycles and loops. Constructing graphs for the training sentences, we could discard graphs with cycles and remove loops with some exceptions, since some of the loops would be justifiable. Upon analyzing such loops, however, we came up with a possible explanation for their presence, which is that the annotators might have failed to find the appropriate type for some events in sentences in the limited controlled vocabulary and would have attempted to use the combination of more than one *component event* to present the event (*merged events*). In Fig. 2, Gene Expression and Positive Regulation events with the event trigger ‘overexpressed’ exemplify such merged events. Most of the other loops would be due to words hyphenating protein mentions and event triggers (e.g., ‘IFNgamma-induced’). We identified the pairs of Gene Expression and Positive Regulation events making loops and then replace them with single merged events.

Finally, we point out two differences between our graph representation and the widely used one proposed by Björne and colleagues [5]. One is that their representation allows only predefined labels of combined event types (e.g., Gene Expression/Positive Regulation), but that our representation allows any possible labels of combined event types. Another is that they do not use merged events, while we evaluate the consequences of these differences, as shown in the Results and Discussion section.

### Statistical model

Given a sentence *x*=(*x*_1_…*x*_*n*_), we constructed graph representations of events by finding the most reliable assignment of labels in a complete directed graph with the words as nodes and removing edges labeled as irrelevant from the graph. We measured the reliability of assignments of labels in terms of output scores of a modified version of a state-of-the-art model proposed by Riedel and McCallum [2], since their model does not allow words with more than one event type. They proposed three models ranging from the simplest one, Model 1, to the most complex one, Model 3. Model 3 was ranked the second in the GENIA Event subtask of the 2011 BioNLP shared task and its variant was ranked the first [12]. However, we developed our model from Model 1 for convenience of experiments, since Model 3 was reported to be much slower than Model 1 in training and predicting.

Given an assignment *L*, our model *M* first checks if the assignment *L* satisfies the following two conditions. One is that identified anchor words (i.e., constituent words of event triggers) have at least one edge labeled with THEME starting from them. Another is that all edges labeled with a role type come from identified anchor words. If the assignment satisfies the conditions, our model assigns scores *M*_*i*,*e*_(*L*_*i*,*e*_|*x*) to all the pairs of an event type *e* and a word *x*_*i*_ (i.e., vertices) and scores *M*_*i*,*j*_(*L*_*i*,*j*_|*x*) to all the pairs of words *x*_*i*_ and *x*_*j*_ (i.e., edges) and take the sum of these scores as the score *M*(*L*) of the assignment *L* as follows:

2 $$ M(L)=\sum_{(i, e)}M_{i, e}(L_{i, e}|x) + \sum_{i, j}M_{i, j}(L_{i, j}|x)  $$

where *L*_*i*,*e*_ takes on a value of either ‘positive’ or ‘negative’, while *L*_*i*,*j*_ takes on a value of either ‘THEME’, ‘CAUSE’ or ‘negative’. Now the extraction of events can be viewed as finding the assignment with the highest score. To find the optimal assignment for a given sentence, we use a modified version of the dynamic programming algorithm proposed by Riedel and McCallum [2]. One may suppose that valid assignments should satisfy other constraints, such as the one that the edge labeled with role types goes to either anchor words or protein mentions. However, such constraints make it hard to efficiently find the optimal assignment of graphs. For this reason, the system first finds the optimal assignment without such constraints, and if the resulting assignment does not contain any cycles, we attempt to refine the assignment so that it satisfies all the constraints. For example, the label ‘negative’ is reassigned to all the incoming edges of a word other than the identified anchor words and protein mentions. When the resulting assignment has a cycle, it does not generate any events for the input sentence.

We scored pairs of a word *x*_*i*_ and an event type *e* using a weight vector *w*_*e*_ as follows:

3a $$ M_{i, e}(positive|x) = w_{e} \cdot \Phi(x_{i}),  $$

3b $$ M_{i, e}(negative|x) = -w_{e} \cdot \Phi(x_{i}),  $$

where *Φ*(*x*_*i*_) is the feature vector of words *x*_*i*_. To define the feature vectors of words, we used their lexical and linear/syntactic contextual information. Lexical information about words is encoded with their surface form, baseform, POS tag and the reliability scores of the entries derivable from them in each event trigger lexicon. The reliability scores are encoded as real-valued features. The linear contextual features of a word (e.g., the word ‘decreased’ in sentence (1)) include center-marked n-grams of words centered around the word (*n* = 2-4) and made out of pairs of baseforms and POS tags (e.g., “decrease:VBN”) and a special symbol ‘PROTEIN’ for protein mentions. For example, the center-marked trigram “either:CC [decrease:VBN] or:CC” is used as a feature for the word ‘decreased’ in sentence (1). They also include the distance from the word to proteins (e.g., “Protein-Distance:5” for the word ‘decreased’ and the protein ‘VDR’ in sentence (1)) and the distance from the word to potential anchor words within the sentences relative to them (e.g., “Trigger-Distance:2” for the word ‘decreased’ and the word ‘increased’ in sentence (1)). The distances of protein mentions are encoded as binary features (i.e., taking either 0 or 1), but features for the distances of potential anchor words take on the maximal reliability score of the corresponding entries in the lexicons. As syntactic contextual features, we encoded their syntactic governors and modifiers (e.g., “number:NNS-MOD(amod)-decrease:VBN” and “numbers:NNS-GOV(dobj)-express:VBP” for the word ‘numbers’ in sentence (1)). Note that these contextual features are intended to exploit words other than anchor words in sensing the event triggers including them.

We also scored the label *L* of a word pair (*x*_*i*_,*x*_*j*_) using a weight vector *w*_*L*_ as follows:

4 $$ M_{i, j}(L|x) = w_{L} \cdot \Phi(x_{i}, x_{j}),  $$

where *Φ*(*x*_*i*_,*x*_*j*_) is the feature vector of a word pair (*x*_*i*_,*x*_*j*_). To define the feature vector of a word pair (*x*_*i*_,*x*_*j*_), we used the following features based on the features used in [1]. Our feature vector consists of the lexical and linear/syntactic contextual features of each of them as defined above, the length of the shortest paths between them and various representations of substructures of paths between them as defined below. First, from a shortest path, we generated the *token sequence* of the pairs of baseforms and POS tags (e.g., “decrease:VBN number:NNS express:VBP” for the word ‘decreased’ and the word ‘express’ in sentence (1), the *dependency sequence* of pairs of the types and directions of dependency relations (e.g., “MOD(amod) GOV(dobj)” for the word ‘decreased’ and the word ‘express’ in sentence (1), and the *token-dependency sequence* of the pairs of baseforms and POS tags and pairs of the types and directions of dependency relations (e.g., “decrease:VBN MOD(amod) number:NNS GOV(dobj) express:VBP” the word ‘decreased’ and the word ‘express’ in sentence (1). From the resulting sequences, we generated n-grams of these sequences (*n* = 2-4).

For efficiency, we assign the label ‘negative’ to those words that do not contain any entry in our event trigger lexicons. We also assigned the label ‘negative’ to edges other than those edges whose starting word contains any entry in the lexicons and whose ending word either refers to a protein or contains an entry in the lexicons for events that take proteins. Since about 98 % of the annotated occurrences in the training corpus contain an entry in the lexicons, this does not incur a large performance penalty but greatly reduces the size and complexity of the problem.

### Learning algorithms

**Fig. 3:**
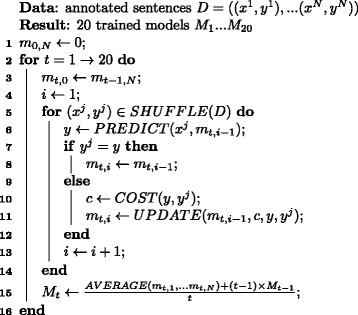
Baseline algorithm

As a baseline algorithm, we used the online prediction-based Passive-Aggressive algorithm [13] with the cost function of penalizing false negative event triggers and edges 3.8 times more heavily than false positive ones as in [2], since the algorithm with this setting successfully trained Model 1 in [2], the one similar to our statistical model. The pseudo-code of this algorithm is shown in Fig. 3. It begins with an initial model with all weights set to 0 (line 1). It takes several passes over the training corpus *D*=((*x*^1^,*y*^1^),...,(*x*^*N*^,*y*^*N*^)), where *x*^*i*^ and *y*^*i*^ are the *i*-th sentence and the *gold-standard graphs* that are automatically derived from the gold-standard event annotations using the Head-Word rule, respectively (line 2). Given a sentence *x*^*j*^, it constructs a graph *y* (line 6) with the help of the current interim model *m*_*t*,*i*−1_. When it makes a mistake (i.e., the predicted graph *y* is not the same as the gold-standard graph *y*^*j*^), we constructed the next interim model *m*_*t*,*i*_, whose output score of *y*^*j*^ goes beyond that of *y*^*j*^ by at least the cost *c* incurred by the mistake, by making a few modifications to the current interim model *m*_*t*,*i*−1_ to keep the knowledge learned so far as much as possible. We take a total of 20 passes over the training corpus, saving the average *M*_*t*_ of the interim models’ weight vectors after each pass (line 15), since the average *M*_*t*_ of interim weight vectors is less likely to over-fit to the training corpus than the individual interim weight vectors as shown by Collins [14]. Here, the total number of passes, that is, 20, was arbitrarily chosen, but it turns out that the number is sufficiently big for learning a statistical model.

**Fig. 4:**
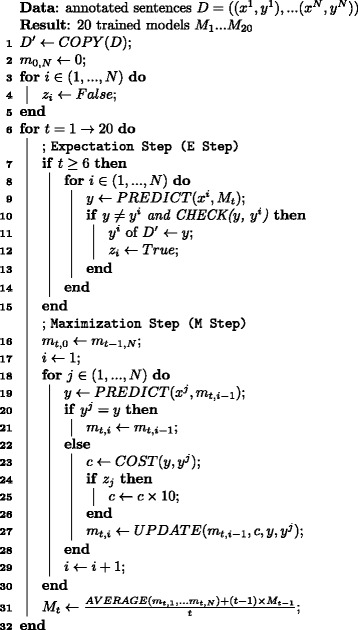
Informed EM algorithms

With a modified version of the baseline algorithm as the M step, we developed the *Informed EM* algorithm, or the EM algorithm with a posterior regularization technique as shown in Fig. 4, where sentences *x* and event annotations *z* are observed and assignments *y* of labels to words and word pairs are missing. Since it would be intractable to enumerate all the possible assignments producing the gold-standard event annotations *z*, we use the Viterbi approximation to the EM algorithm under the unreasonable assumption that the most probable assignment has a remarkably higher probability than the second probable assignment. This case may also have the counterpart of the Inside-Outside algorithm, or the efficient implementation of the EM algorithm widely used in learning PCFGs in an unsupervised manner, but we leave the design of such an algorithm for future research. To incorporate the gold-standard annotations into the EM algorithm, we impose constraints on possible assignments, which are derived from the gold-standard annotations.

Now we describe the pseudo-code of this algorithm as shown in Fig. 4. We constructed the adjusted annotation set *D*^′^, where the adjusted graphs *y*^*i*^ are initially their corresponding gold-standard graphs (line 1). It takes several rounds (line 6), but behaves like the conventional EM algorithm of alternatively applying the E and M steps after the first five rounds (line 7). Here, the number of rounds for initialization, that is, five, was arbitrarily chosen. Since the EM algorithm may converge models into local optima, we need to take care of initial models with which the EM algorithm begins. During the first five rounds, we trained the model by applying only the M step in a supervised learning manner similar to that of the baseline algorithm, since the resulting model would be closer to the true model, if it exists, than randomly constructed models. In the E step, it predicts a graph *y* for a sentence *x*^*i*^ with the current interim model *M*_*t*_ (line 8). It sets the adjusted graph *y*^*i*^ to the prediction *y* if the prediction *y* is not matched with the current adjusted graph *y*^*i*^ and satisfies predefined constraints (lines 10 and 11). To enforce models to predict anchor words other than the head words of the annotated event triggers, we modify the cost function to penalize errors for sentences with updated graphs 10 times more severely than for the others as in domain adaptation studies (e.g., [15]) (lines 24-26).

We came up with the following constraints. One is the *basic constraint* that the adjusted graph should encode the same event types and argument types as the gold-standard graphs. For example, if a Positive Regulation event with a Gene Expression event as THEME appears in the gold-standard annotations, this constraint requires that one or more Positive Regulation and Gene Expression events appear in the adjusted graphs and that the Positive Regulation events should take a Gene Expression event, but does not take care of their event triggers. Another is the *confidence constraint* such that the percentage difference in output scores between candidate graphs *y* for next adjusted graphs and current adjusted graphs *y*^*j*^ should be equal to or greater than the *confidence constraint constantα*. To reflect the gold-standard annotations more faithfully, we come up with the *non-overlapping constraint* (*NOC* for short) that two event triggers with the same event type in gold-standard graphs cannot be mapped into a single word in their corresponding adjusted graphs. For example, consider sentence (5).

(5) The c-jun mRNA, which is constitutively **expressed** in human peripheral-blood monocytes at relatively **high levels**, was also slightly **augmented**... (PMID:1313226)

The words “high levels” and ‘augmented’ indicate the presence of two distinct Positive Regulation events with the same Gene Expression event with the event trigger ‘expressed’ as THEME. Since they indicate the presence of events of the same type with the same participants, those assignments where only one of the words is labeled with ‘Positive Regulation’ violate the non-overlapping constraint, but not the first two constraints.

Since there might be cases when the event triggers of more than one event of the same type can be merged without any problems but when the non-overlapping constraint prohibits any merging, we came up with a more relaxed constraint, or the *distance constraint* that the distance between event triggers in candidate graphs *y* for next adjusted graphs and event triggers with the same event type in current adjusted graphs *y*^*j*^ (e.g., the distance between ‘levels’ and ‘augmented’ is four) should be equal to or less than the distance constraint constant *β*. In sentence (5), those graphs without any one of the event triggers of these two Positive Regulation events would also violate the distance constraint with *β*≤3.

## Results and Discussion

We used the baseline and informed EM algorithms to train our statistical models and evaluated the models on the development corpus with respect to standard evaluation metrics, such as recall, precision and F-score.

### Evaluation of proposed graph representations

To measure the consequence of the substitution of single merged events for Positive Regulation and Gene Expression events sharing single words, we reconstructed training event annotations by converting the gold-standard annotations into graphs and the resulting graphs into event annotations, since events that cannot be encoded in graphs cannot survive in the reconstruction process. The substitution may decrease the number of events removed after the reconstruction process but may increase the number of incorrect events generated by the graph-to-event conversion algorithm after the reconstruction process, leading to changes of the F1-score of the reconstruction event annotations on the original event annotations. We measured the F1-score of the reconstructed event annotations twice, one with the substitution and another without the substitution. We found that the substitution leads to an increase in the F1-score of the reconstructed event annotations by 1.13 % points, and in particular for Positive Regulation events by 3.14 % points, though the F1-score for Gene Expression events is slightly decreased by 0.06 % points.

**Fig. 5:**
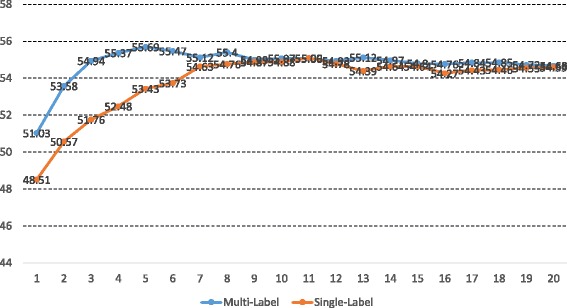
Comparison Between Multi-Label and Single-Label Statistical Models. Each point (*x*,*y*) indicates that a model trained by taking *x* rounds has an F-score of *y*. These models are trained using the baseline algorithm

We also measured the consequences of allowing words with more than one event type. We used the baseline algorithm to train multi-label and single-label statistical models. We found that the multi-label models outperform the single-label models most of the time as shown in Fig. 5. To evaluate the statistical significance of the superiority of the multi-label models over the single-label models, we carried out the one-tailed paired Student’s t-test for the pairs of the two points with the same x value. The reason for the use of the one-tailed test, but not the two-tailed test, is that only one direction (multi-label models’ scores > single-labeled models’ scores) is considered to be against the null hypothesis that the multi-label models are not superior to the single-labeled models. According to the test, the superiority of the multi-label models over the single-label models is shown statistically significant with a *p*-value of 0.0013. After the first five rounds, the more rounds we took to train models the lower performance the resulting models showed. This would be because the models trained by taking many rounds are likely to over-fit to the training corpus. We suggest to stop the learning process of models when the models’ performance on a held-out corpus starts to decrease.

Table 1 shows the summary of the performance of the models of each type. Since the Informed EM algorithm applies the E step after the first five rounds, to be fair with the Informed EM algorithm, we calculate averages and sample standard deviations of the F-scores of the models trained by taking more than five passes.

**Table 1:** Performance of multi-label and single-label statistical models. These models are trained using the baseline algorithm

	Single-label (R/P/F)	Multi-label (R/P/F)
BEST	46.8/67.0/55.1	47.3/67.7/55.7
AVG.	46.2/66.6/54.6	46.6/67.1/55.0
(STD.)	(0.36/0.41/0.32)	(0.23/0.21/0.30)

The single-label models are in fact our implementation of Model 1 of Riedel and McCallum [2], which was reported to have the F1-score of 56.2 % for the development corpus, and the best has a similar F-score of 55.1 %, where the difference may be due to implementation details regarding the feature vector construction.

### Evaluation of the informed EM algorithm

**Fig. 6:**
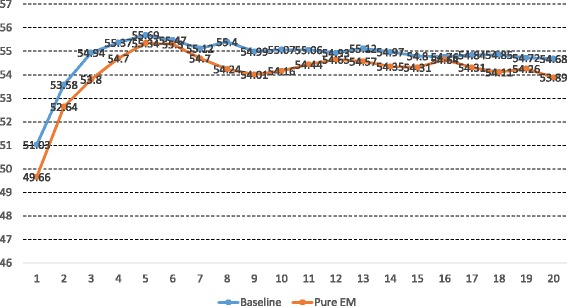
Comparison Between the Baseline and Pure EM Algorithm. Each point (*x*,*y*) indicates that a model trained by taking *x* rounds has an F-score of *y*

To examine the effect of the posterior regulation, we first use the Informed algorithm without any constraints (the *pure EM* algorithm) to train models. It is again unsurprising that the more rounds we took to train models the lower performance the resulting models showed as shown in Fig. 6. As a result, the best one is the model it took six passes to train, which shows a recall of 47.12 %, a precision of 67.04 % and an F-score of 55.34 %.

At the first E step, more than a thousand of adjusted graphs were updated and at subsequent E steps, fewer than half a hundred graphs were updated, suggesting that the models are converging (the total number of sentences is about seven thousands) and the pure EM algorithm would have trained models to predict similar but unintended graphs.

We evaluated our Informed EM algorithm with various constraint sets, all of which include the basic constraint, as shown in Tables 2 and 3. The comparison between Table 1 on the one hand and Tables 2 and 3 on the other shows that most models outperform models trained by the baseline algorithm in terms of both the best and averaged F-scores. To assess the statistical significance of their superiority over the models trained with the baseline algorithm, we calculate *p*-values with respect to the one-tailed paired Student’s t-test for the pairs of models trained by taking the same number of rounds, as shown in Table 4.

**Table 2:** Best performance of informed EM models

*α*=	*β*=2 (R/P/F)	*β*=100 (R/P/F)
Without NOC		
0.1	48.0/68.2/56.3	47.6/68.3/56.1
0.2	47.6/68.6/56.2	47.4/68.5/56.0
0.3	47.7/68.8/56.3	47.3/67.5/55.7
0.4	47.1/67.8/55.6	47.6/67.7/55.9
With NOC		
0.1	47.3/68.9/56.1	47.5/68.1/55.9
0.2	47.3/68.0/55.8	47.5/**69.3**/56.4
0.3	**48.1**/68.9/**56.7**	47.2/68.1/55.8
0.4	46.8/68.9/55.8	47.3/67.7/55.7

The best figures are set in bold-face

**Table 3:** Average performance of informed EM models

*α*=	*β*=2 (R/P/F)	*β*=100 (R/P/F)
Without NOC		
0.1	**47.9**/66.8/55.8	47.3/67.7/55.7
	(0.27/0.56/0.31)	(0.22/0.30/0.23)
0.2	47.1/68.0/55.7	47.1/68.1/55.7
	(0.35/0.86/0.42)	(0.22/0.21/0.16)
0.3	47.4/67.9/55.8	47.3/66.8/55.4
	(0.18/0.39/0.23)	(0.13/0.22/0.13)
0.4	46.7/67.5/55.2	47.0/67.7/55.5
	(0.38/0.52/0.21)	(0.35/0.23/0.30)
With NOC		
0.1	46.9/68.0/55.5	47.1/67.6/55.5
	(0.23/0.39/0.26)	(0.15/0.23/0.16)
0.2	47.1/67.6/55.5	47.2/68.3/55.8
	(0.22/0.29/0.20)	(0.22/0.65/0.35)
0.3	47.6/68.0/**56.0**	47.0/67.1/55.3
	(0.38/0.45/0.40)	(0.27/0.36/0.29)
0.4	46.5/**68.4**/55.4	47.1/67.6/55.5
	(0.33/0.72/0.42)	(0.24/0.39/0.22)

The best figures are set in bold-face and the sample standard deviations are bracketed

**Table 4:** *p*-values for informed EM models

*α*=	*β*=2 (w.o/w NOC)	*β*=100 (w.o/w NOC)
0.1	3.32E-09/1.86E-04	1.03E-06/4.47E-06
0.2	9.98E-07/1.21E-08	3.58E-09/1.05E-08
0.3	**9.59E-12**/3.93E-09	4.38E-06/2.95E-03
0.4	4.37E-02/1.19E-04	2.50E-08/6.70E-07

The best figures are set in bold-face

We analyzed the effect of the choice of constraints on the performance of models. The high confidence constraint constant *α* reduces the number of updates in the adjusted graphs, making the resulting models similar to models trained by the baseline algorithm as shown in Table 5. The distance constraint (*β*=2) reduces the number of updates in the adjusted annotation set and for most times increases the best F-scores but not the averaged F-scores. The non-overlapping constraint also reduces the number of updates but not always increases the best and averaged F-scores. Note that, even though our best model is the model we trained with the non-overlapping constraint, the best combination of constraints would be with the *α* value of 0.3 and the *β* value of 2 and without the non-overlapping constraint as indicated in Table 4.

**Table 5:** Updated graphs for informed EM models

*α*=	*β*=2 (w.o/w NOC)	*β*=100 (w.o/w NOC)
0.1	72/47	98/50
0.2	34/18	46/31
0.3	16/11	25/15
0.4	9/8	9/5

Finally, we chose the best baseline model (a multi-labeled model) and best proposed model (*α*=0.3, *β*=0.2, no use of non-overlapping constraint) in terms of the performance on the development corpus and evaluated them on the BioNLP’09 test corpus. We found that the best baseline model has a recall of 42.2 % and a precision of 65.5 % and F-score of 51.3 %, while the best proposed model has a recall of 42.2 % and a precision of 66.4 % and F-score of 51.6 %, suggesting that the proposed models slightly outperform the best baseline model.

### Analysis of adjusted graphs

We observed updates of shifting the mark of anchor words from empty words into content words (e.g., ‘activity’ vs. ‘-binding’ in the noun phrase ‘DNA-binding activity’ (PMID:9115366)) and from words distant from the participants of the anchored events into words closer to them (e.g., ‘simulates’ vs. ‘activation’ in the phrase “simulates the activation of” (PMID:8557975)). There were also updates of labeling more than one words as the event trigger of an identical event (e.g., ‘results’ and ‘increases’ in a phrase starting with “results in increases of” (PMID:2121746)).

Unexpectedly, we found that sets of edges were updated more often than the position of anchor words. Some edges were copied and redirected (e.g., copies of all edges coming from ‘results’ are attached to ‘increase’ in the preceding example), leading to new events whose presence makes sense, where some of them have corresponding existing ones and some of them are completely new. For example, a Regulation event of granulocyte-macrophage colony-stimulating factor was created on sentence (6) with an annotated Regulation event of the Expression event of the protein.

(6) **Regulation** of **granulocyte-macrophage colony-stimulating factor** and E-selectin **expression** in endothelial cells by cyclosporin A and the T-cell transcription factor NFAT. (PMID:7545467)

Some edges not used in deriving events from the graphs are removed, leading to the removal of events that seem to be inferred. For example, sentence (7) below has an annotated Positive Regulation event of H2 receptors, which was removed by an update. The rationale behind this annotation is that a sensible way of using H2 receptors to increase cAMP and c-fos expression is to activate H2 receptors.

(7) Histamine transiently **increased** cAMP and c-fos expression through **H2 receptors**. (PMID:9187264)

Of course, there are inexplicable updates. Table 6 shows the distribution of types of 16 updates occurring in learning the best proposed model (*α*=0.3, *β*=0.2, no use of non-overlapping constraint). It shows that there are various types of ambiguity, even though the algorithm finds a small number of each type of cases.

**Table 6:** Distribution of types of 16 updates

Description	Count
Adding events similar to existing ones	7
Adding missing but reasonable events	4
Shifting the mark of anchor words	2
Removing duplicated and inferred events	2
Wrongly adding an incorrect event	1
Total	16

## Conclusion

In this study, we looked into the possibility that adjustments to the annotated span of event triggers to reduce inconsistencies across them lead to an improved performance of event extraction systems. In order to make adjustments automatically in favor of statistical models, we developed an Informed EM algorithm, or the EM algorithm with a posterior regularization technique that exploits the gold-standard event trigger annotations in the form of constraints. The algorithm (the best F-score=56.7 %) is shown to outperform our baseline algorithm (the best F-score=55.7 %) on the BioNLP’09 development corpus in a statistically significant manner (*p*-value=9.59E-12), indicating that proper adjustments of event trigger annotations would be beneficial. Our algorithm (F-score=51.6 %) is also shown to slightly outperform our baseline algorithm (F-score=51.3 %) on the BioNLP’09 test corpus. The annotations generated by the algorithm indicate that there are various types of ambiguity in event annotations including ambiguity in the span of event triggers, even though the algorithm finds only a small number of such cases.

However, there are still remaining issues. First, we use the Viterbi approximation to the EM algorithm, whose soundness is not well grounded. We anticipate that there would be a counterpart of the Inside-Outside algorithm, an efficient implementation of the EM algorithm used in learning PCFGs. Second, we fixed the parameters *α* and *β* as confidence and distance constraint constants, respectively, during training models. However, Smith and Eisner [16] show that it would be beneficial for the EM algorithm guided by prior knowledge to soften the constraints, as model parameters are converging. We anticipate that such update scheduling would also be beneficial for the informed EM algorithm. Third, we applied this approach only to the 2009 BioNLP shared task. Since this approach is not specific to this task, there is a possibility of applying this approach successfully to similar tasks, such as Infectious Disease (ID) and Epigenetic and Post-translational Modification (EPI) tasks defined in the 2011 Bio-NLP shared task. We plan to address these issues in the future.
